# Improving the prediction of organism-level toxicity through integration of chemical, protein target and cytotoxicity qHTS data†
†Electronic supplementary information (ESI) available. See DOI: 10.1039/c5tx00406c


**DOI:** 10.1039/c5tx00406c

**Published:** 2016-03-03

**Authors:** Chad H. G. Allen, Alexios Koutsoukas, Isidro Cortés-Ciriano, Daniel S. Murrell, Thérèse E. Malliavin, Robert C. Glen, Andreas Bender

**Affiliations:** a Centre for Molecular Informatics , Department of Chemistry , Lensfield Road , Cambridge CB2 1EW , UK . Email: ab454@cam.ac.uk ; Tel: +44 (0)1223 762983; b Unité de Bioinformatique Structurale , Institut Pasteur and CNRS UMR 3528 , Structural Biology and Chemistry Department , Paris , France; c Department of Surgery and Cancer , Faculty of Medicine , Imperial College London , Sir Alexander Fleming Building , South Kensington Campus , London SW7 2AZ , UK

## Abstract

Prediction of compound toxicity is essential because covering the vast chemical space requiring safety assessment using traditional experimentally-based, resource-intensive techniques is impossible. However, such prediction is nontrivial due to the complex causal relationship between compound structure and *in vivo* harm. Protein target annotations and *in vitro* experimental outcomes encode relevant bioactivity information complementary to chemicals’ structures. This work tests the hypothesis that utilizing three complementary types of data will afford predictive models that outperform traditional models built using fewer data types. A tripartite, heterogeneous descriptor set for 367 compounds was comprised of (a) chemical descriptors, (b) protein target descriptors generated using an algorithm trained on 190 000 ligand–protein interactions from ChEMBL, and (c) descriptors derived from *in vitro* cell cytotoxicity dose–response data from a panel of human cell lines. 100 random forests classification models for predicting rat LD_50_ were built using every combination of descriptors. Successive integration of data types improved predictive performance; models built using the full dataset had an average external correct classification rate of 0.82, compared to 0.73–0.80 for models built using two data types and 0.67–0.78 for models built using one. Pairwise comparisons of models trained on the same data showed that including a third data domain on top of chemistry improved average correct classification rate by 1.4–2.4 points, with *p*-values <0.01. Additionally, the approach enhanced the models’ applicability domains and proved useful for generating novel mechanism hypotheses. The use of tripartite heterogeneous bioactivity datasets is a useful technique for improving toxicity prediction. Both protein target descriptors – which have the practical value of being derived *in silico* – and cytotoxicity descriptors derived from experiment are suitable contributors to such datasets.

## Abstract 2

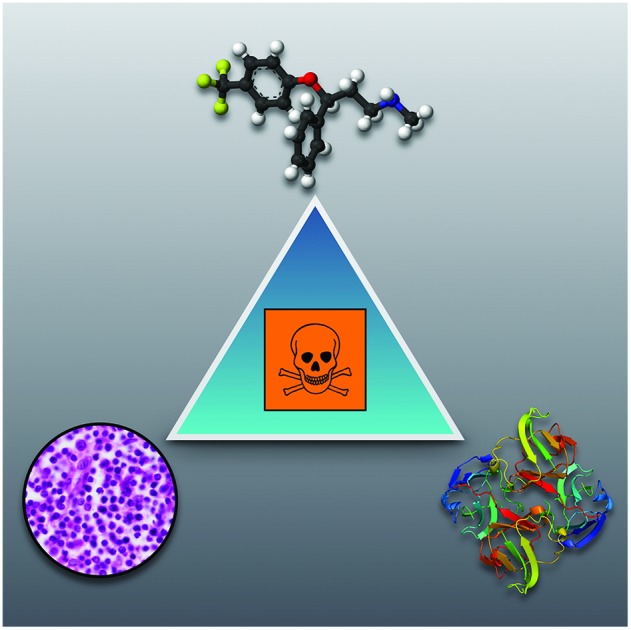
Using three descriptor domains – encoding complementary bioactivity data – enhances the predictive power, applicability, and interpretability of rat acute-toxicity classifiers.

## Introduction

Toxic substances are those “which, if they are inhaled or taken internally or if they penetrate the skin, may involve serious, acute or chronic health risks and even death”.^1^ Contemporarily, the Globally Harmonized System of Classification and Labelling of Chemicals (GHS) gives globally accepted definitions for different types of toxicity including acute toxicity, carcinogenicity and specific organ toxicity.^2^ Toxicology, the study of toxicity, is concerned both with the evaluation of toxicity in substances, and the elucidation of their toxic mode-of-action.^3^ Toxicology is of particular importance to medicinal chemistry, as the discovery of toxic side effects late in the drug discovery process is dangerous to study volunteers and patient groups, as well as being highly wasteful as drug development must be abandoned after significant cost; understanding the nature and causes of such toxicity enables researchers to minimize the likelihood of uncovering adverse compound effects late in the process. In addition, many other areas as diverse as agrochemistry, consumer goods, and advanced materials would benefit from a better understanding of the toxicity associated with chemical structures.

Traditional toxicology has focused on extrapolating human toxicity from animal toxicity, based on the assumption that adverse toxic reactions in animals imply the potential for similar consequences in humans – an assumption that often does not hold true.^4^,^5^ Experimental animal testing *in vivo* is also time- and resource-expensive, and usually low-throughput.^6^ Alongside such practical and economic reasons to seek alternative methodologies, there are also social and legal pressures to minimize the use of laboratory animals, such as the recent ban on testing cosmetic products on animals in the EU.^7^ In addition, there is a broad movement toward more precautionary governmental treatment of potential toxicological risks.^8^,^9^ Reliable and efficient methods of predicting organism-level toxicity using data which has already been obtained are therefore of great utility.

Computational toxicology is a growing discipline which pursues the goal of predicting compounds’ toxicological effects *in silico*.^10^,^11^ This goal has become attainable due to recent advancements in three areas: (i) technological improvements affording high-throughput data generation techniques, *e.g.* high-throughput screening (HTS) assays, and the accumulation of their results in publicly-accessible repositories;^12^ (ii) the development of advanced machine-learning algorithms, which are able to find relationships within large databases of chemical and biological data;^13^ and (iii) the increasing availability of powerful computational resources capable of applying these algorithms to large datasets.^14^ The combination allows the development of quantitative structure–activity relationship (QSAR) models able to predict toxicity from compound structure.^15^

HTS was originally developed by the pharmaceutical industry as a technique to identify potential drug leads from large panels of candidate molecules.^16^ Traditionally, the HTS assays were performed for single compound concentrations; those chemicals that gave a response could then be screened again at a range of concentrations. The initial single data point screening leads to a high proportion of false positives and false negatives; this is acceptable when seeking one lead from many candidates; however, this is less acceptable when screening for toxicity because false negatives are less tolerable, and because more subtle biological interactions go undetected.^17^ The quantitative HTS (qHTS) paradigm,^18^ wherein all compounds are screened for a concentration-dependent response from the start, allows for a more nuanced assessment of biological activity while retaining the advantage of inexpensive high throughput. Furthermore, the presence of multiple data points enables the identification of errors outlying the curve which would not be detected in single-point screening. Consequently, this paradigm produces data highly suitable for large-scale toxicity modelling.^19^

The development of novel experimental technologies has been followed by many coordinated endeavours to produce large-scale databases of the resulting data in order to prioritize future toxicological evaluation of compounds.^20^,^21^ Novel data sources and repositories afford increasing opportunities to study the complex biological effects of chemicals, and enable the introduction of heterogeneous data sources in broad-scale predictive modelling.

A further result of the dramatic increase in availability of such large-scale bioactivity data is the development of computational models that can predict the protein targets of a ligand given its chemical structure.^22^ These models are founded on the assumption of the similarity principle, *i.e.* that chemicals of similar structures will exhibit similar activities; in the frame of target prediction models, this assumption is natural as it is equivalent to the assumption that similar molecules will bind to the same active site, or otherwise modulate the same targets’ activities. However, target prediction transcends a simple similarity search due to the flexibility of binding pockets, and the possible presence of allosteric sites, affording interactions with chemically quite dissimilar compounds. Machine learning methods are thus necessary to capture the complex, non-linear relationships between chemical and biological spaces.

The recent easy availability of bioactivity and structural data, and the heterogeneous nature of this data, now permits its utilisation in the prediction of organism-level phenotypes such as toxicity. Hence, *in silico* prediction of biological activity is no longer solely performed using the traditional QSAR paradigm of extrapolation from *structural* chemical descriptors alone; rather there have been a number of studies exploring the additional use of descriptors derived from cell-line exposure response.^23^,^24^ Such integration of descriptors from heterogeneous data domains provides more accurate predictions, due to the variety of complementary input data. Indeed, because the chemical responsible for a toxicity may only be generated through biotransformation of the substance to which the organism has been exposed, data from *in vitro* experiments which include metabolic competence may be required to detect the toxicity.^25^

The present work builds upon the study of Sedykh *et al.*^26^ in which chemical descriptors in combination with qHTS-derived descriptors were used to predict rat acute toxicity. The authors found that the inclusion of qHTS-derived descriptors enhanced both the predictive performance and the applicability domain coverage of their models – once a suitable noise-filtering algorithm had been applied to the qHTS data. They stated that their results “provide compelling support for increasingly sophisticated and tailored predictive approaches that incorporate all available information (chemical, biological, and concentration–response) in modeling”.

In this study, we make use of the data collated and noise-filtered by Sedykh *et al.*,^26^ and additional include protein-target affinity scores to generate a triply heterogeneous dataset. The novelty of the work arises from the use of a validated *in silico* target-prediction algorithm to facilitate the creation of a dataset comprising three data domains suitable for the prediction of *in vivo* toxicity.

Compounds’ toxicity can arise for a variety of reasons. The physiochemical properties of the whole molecule (which may result in adverse interactions with *e.g.* cell membranes), specific functional groups of undesirable reactivity, and the ability of the compound to bind to protein targets can all have toxic consequences. As toxicity can be caused by multiple properties, so multiple data domains describing encoding these properties may be required for the prediction of these toxicities.^11^

Seven sets of classification models for the prediction of rat toxicity have been developed using varying combinations of the three data domains outlined above; the predictive power of the models is used to illustrate the power of heterogeneous data integration.

## Materials and methods

### Data sources

The experimental data used in the work was previously used in the study of Sedykh *et al.*^26^ in which quantitative high-throughput-screening (qHTS) data were combined with molecular descriptors in the prediction of acute rat toxicity.

The qHTS data were generated by the National Toxicology Program and originally extracted from PubChem's BioAssay Database.^27^ These comprise concentration–response profiles for 1408 substances against 13 rat, mouse and human cell lines;^28^ the concentration of substances varies between 0.6 to 92 μM and the response values correspond to the decrease in cell viability compared to controls. These data were curated by Zhu *et al.*^24^ to eliminate duplicates, and filtered for noise by Sedykh *et al.*^26^ using the parameters determined to be optimal in their study.

The acute rat toxicity data were collected and curated by Zhu *et al.*^29^ The toxicities of 7385 unique compounds were expressed as the negative logarithm of the median lethal dose, –log_10_ (LD_50_/mol kg^–1^) or pLD_50_.

Within the set of 695 structures for which both acute rat toxicity data and qHTS data were available, in accordance with the previous procedure of Sedykh *et al.*,^26^ substances with a toxicity pLD_50_ < 2 were classified as nontoxic, those of pLD_50_ > 3 were classified as toxic, and the remaining chemicals of 2 < pLD_50_ < 3 were classified as marginal and therefore discarded. Compounds classed as toxic correspond to acute toxicity categories 1–3 of the GHS, and non-toxic compounds to category 5.^2^

The result was a dataset of 367 structures, each one being accompanied by 13 noise-filtered qHTS profiles. 275 of the compounds were classed as non-toxic and 92 were classed as toxic (this class imbalance will be addressed in the modelling procedure). Further information on the dataset's chemistry is given in the ESI.†

### Descriptor generation

An overview of the descriptors used in this study is given in Table 1. The procedure for generating them is outlined in the following section.

**Table 1:** Overview of descriptor sets from the chemical, protein target, and cytotoxicity domain to be used in modelling toxicity data in all possible combinations. In each modelling repeat, the feature selection and pre-processing procedure was applied to the data in the respective modelling set to select an optimum similarly sized subset of descriptors from each domain

Data domain	Details	Source	Information encoded
Chemical	192 2D descriptors	MOE	Chemical structure and physiochemical properties
Protein target	477 human target-affinity descriptors	*In silico* algorithm trained on dataset extracted from ChEMBL version 14	Translation of chemical space into biological space; likelihood of interaction with subset of human proteome
Cytotoxicity	182 dose–response datapoints of 14 concentrations across 13 human, rat and mouse cell lines, scaled such that the maximum response for each curve equals 1.	Original data extracted from PubChem and processed to remove noise as per study of Sedykh *et al.* (2011)	Experimental cell-viability outcomes of compound exposure

After standardization (see ESI† for details), Molecular Operating Environment (MOE) (version 2013.08, 2013, Chemical Computing Group Inc.) was used to calculate 192 2D molecular descriptors for each structure.

An in-house developed *in silico* algorithm,^30^ which has found a number of applications in recent studies,^31^,^32^ was used to generate protein target affinity descriptors for each chemical from its standardized structure. The training set for the target prediction model was extracted from ChEMBL version 14, comprising over 10 million bioactivities covering 9003 targets and over a million distinct compounds, all derived from the primary literature.^33^ To be included in the training set, compounds had to have a *K*_i_, *K*_d_, IC_50_ or EC_50_ of at least 1 μM against human protein targets; compound-target associations had to have an assay-to-target confidence score of 8 or 9 (corresponding to a single protein assignment, either directly to a homologue); and target classes had to be associated with at least 50 data points. This left 477 human protein target classes that the algorithm was able to predict. The compounds were stored as SMILES and were converted to circular Molprint2D descriptors,^34^,^35^ implemented using the open-source Open Babel package.^36^ The target prediction algorithm uses an implementation of the Laplacian-Modified Naïve Bayesian Classifier.^37^ For this study, in order to generate descriptors suitable for toxicity prediction, each compound was annotated with a measure of its Bayesian likelihood of interaction (also called its “score”, and corresponding to the parameter which is ordinarily used to determine class membership by Bayesian classifiers) for every protein in the model: these scores were used as the protein target descriptors. It is important to note that the numerical value of these descriptors corresponds to the expectation of an interaction, rather than quantitatively predicting the affinity (*e.g. K*_i_) or potency (*e.g.* IC_50_) of any such interaction.

The qHTS profiles comprised 14 concentration–response values across 13 cell lines. Each of the 13 sets of concentration–response variables for each molecule was scaled such that the maximum response was unity, producing 182 cell line-concentration point cytotoxicity descriptors.

Due to the three descriptor domains used in this study, the total number of generated descriptors is comparable to the total number of molecules in the dataset. It is generally acknowledged that an excessive number of descriptors is undesirable in a QSAR model,^15^ notwithstanding that certain modelling algorithms (*e.g.* partial least squares) reduce the dimensionality of the problem by *e.g.* considering the variance of the data as principle components. Furthermore, unequal numbers of descriptors from each data type may allow one type to dominate the model due to the random sampling inherent in the random forests algorithm. To overcome this, before modelling, maximum permissible descriptor correlation values were derived for each domain independently; these values were used during the pre-processing routines to reduce each descriptor set to a similar size (see ESI† for details). Alternative techniques to reduce the number of descriptors (such as information gain analysis and genetic algorithms) were explored, but were not found to afford an increase in performance to compensate for the increase in computational burden.

The experimental data and descriptors used in this study are available for download at the University of Cambridge data repository *via* repository.cam.ac.uk.

### Modelling workflow

**Fig. 1:**
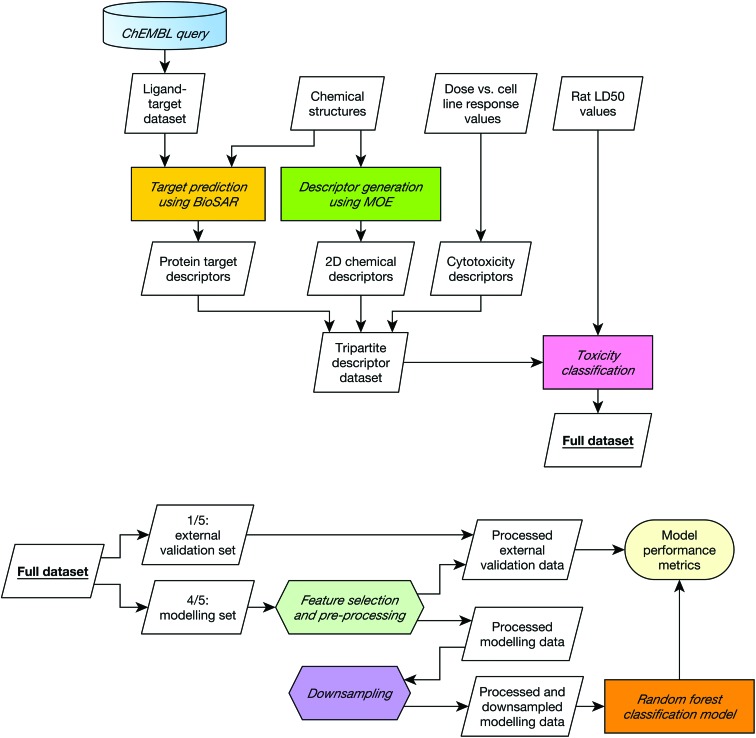
Data collation (top) and modelling (bottom) workflows. The modelling workflow was repeated five times, varying which fifth of the data was held out as the external validation set such all data was included in the external set once. This five-fold cross validation was repeated 20 times with 20 different 5-way cross-validation splits. The entire procedure was repeated for each combination of descriptors in order to establish whether combining different input domains improve toxicity predictions on the dataset used in this study.

A diagrammatic overview of the workflow used in this study is given in Fig. 1.

The model was implemented in R using the caret package.^38^ The metrics used to assess model performance were sensitivity, selectivity and the correct classification rate (CCR). Here, sensitivity is the fraction of correctly identified toxic compounds; selectivity is the fraction of correctly identified non-toxic compounds; and CCR the mean of the two. The performance of 100 models was recorded; these 100 models were generated using twenty 5-fold external cross validation procedures such that twenty toxicity predictions are made for each compound.

In 5-fold cross-validation, the whole dataset is divided into five subsets, and each subset is held out from the rest of the data. Models are trained using the data from the remaining four subsets as the modelling set and tested using the hold-out subset as the external validation set. The procedure is performed five times holding out a different subset each time. Class stratification ensures that the ratio between classes is consistent across subsets.

To ensure a true external assessment, for each round of cross-validation the external validation set was removed from the remaining data before any pre-processing. All pre-processing transformations were therefore determined solely from the structures present in the modelling set and then applied uniformly to both the modelling set and the external validation set.

The following pre-processing procedure was applied to every modelling set before model building, and to each descriptor type independently.

1. Descriptors of zero and near-zero variance were discarded.

2. The domain-specific correlation cutoffs were applied.

3. The descriptors were range scaled to vary between 0 and 1.

4. A downsampling routine was performed, which discarded those nontoxic compounds over a certain distance in chemical space from toxic compounds to afford an approximately class-balanced dataset (see the ESI† for details of the routine).

A random forest classification model was trained on the resultant final modelling set using the following parameters: *n* (the number of trees) = 500, and *mtry* (the number of variables to be randomly chosen for each node) = the square root of the total number of descriptors. The predictive accuracy of the resultant model was assessed by measuring the sensitivity, selectivity and CCR on the held-out subset.

This procedure was performed five times in each cross-validation round, and the 5-fold cross validation itself was performed 20 times with different five-way splits. The entire 20-repeat 5-fold cross-validation routine was performed for each possible combination of descriptor types. To ensure fair comparison, the same random, class-stratified splits were re-used for assessing the performance of each descriptor combination.

### Model robustness

*y*-Scrambling (also called *y*-randomization) was used to ensure the performance of each model could not be the result of chance over-fitting.^39^ For each of the 100 models built using each combination of descriptors 10 similar models were trained on scrambled data, their performances recorded, and a one-tailed *t*-test performed to measure the probability the model's performance falls within the distribution of scrambled models.

## Results and discussion

### Effect of data domains on model performance

**Fig. 2:**
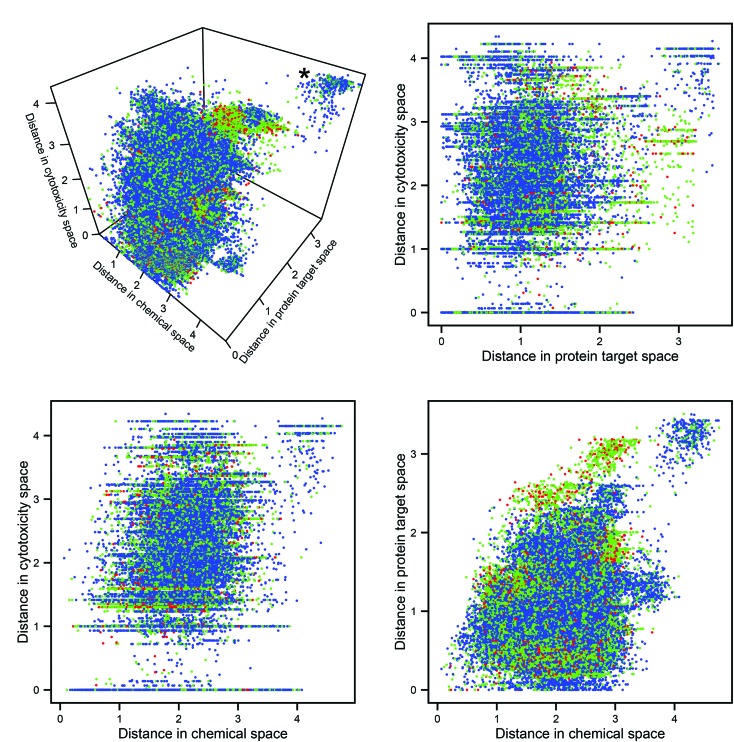
Pairwise Euclidean distances in the three descriptor spaces. Each point represents a pair of compounds: blue points represent non-toxic pairs, red points represent toxic pairs, and green points represents pairs which differ in toxicity. The plots allow for inspection of the relationship between the three data domains and toxicity. The cluster representing dissimilarity in every space (marked with an asterisk on the 3D plot) does not contain any toxic pairs.

The Euclidean distances in the three descriptor spaces of all pairwise combinations of molecules are plotted in Fig. 2 (in all figures utilizing descriptor space, the domain-specific correlation cutoffs were applied over the whole dataset). The cluster at the rear top-right of the 3D plot represents pairs of compounds dissimilar in every data domain. It can be seen that this cluster contains no toxic pairs, and that therefore no two toxic substances are entirely dissimilar in all three descriptor domains. It can also be seen that, outside of the aforementioned cluster of diverse chemicals, pairs which are distant in protein space tend to consist of at least one toxic compound; this may be rationalized through the argument that substances which have diverse protein targets may comprise either a highly biologically active (and potentially toxic) substance and a non-active partner, or else two substances having diverse biological activities (both potentially harmful).

Mantel tests were performed to assess the correlation between pairs of distance matrices within these domains: no pair produced a correlation coefficient >0.29, indicating a great degree of linear independence. The expectation that chemical, protein target, and cytotoxicity descriptors encode *different* information about compound bioactivity is therefore corroborated; however, whether this different information has relevance for the improved prediction of toxicity had to be investigated in the next step.

**Fig. 3:**
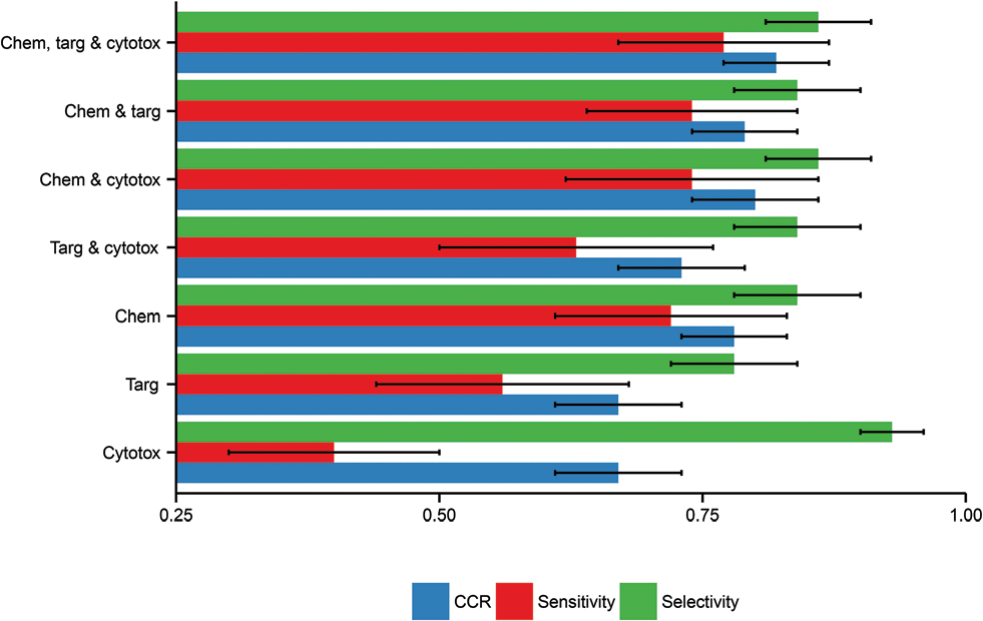
Performance distributions of predictive model build using each combination of descriptor domains. There is a clear trend toward better performance with increased data inclusion. Here error bars are used to display the standard deviations in the performance distributions, illustrating the marked deviation from the mean in several cases. Abbreviations: Chem, chemical descriptors; Targ, protein target descriptors; Cytotox, cytotoxicity descriptors.

Here, the performance of the models built in this study further substantiate this hypothesis, inasmuch as models built using more data domains tend to have improved performance. The distributions of these performance metrics are given in Table 2 and visualized in Fig. 3.

**Table 2:** Distributions of performance metrics for models built using each combination of data. Although the mean performance is improved through increasing data integration, the standard deviations indicate strong variability of performance across different selections of training and test data

Descriptor domains	CCR (mean ± SD)	Sensitivity (mean ± SD)	Selectivity (mean ± SD)
Chemical only	0.78 ± 0.05	0.72 ± 0.11	0.84 ± 0.06
Protein target only	0.67 ± 0.06	0.56 ± 0.12	0.78 ± 0.06
Cytotoxicity only	0.67 ± 0.06	0.40 ± 0.10	0.93 ± 0.03
Chemical and protein target	0.79 ± 0.05	0.74 ± 0.10	0.84 ± 0.06
Chemical and cytotoxicity	0.80 ± 0.06	0.74 ± 0.12	0.86 ± 0.05
Protein target and cytotoxicity	0.73 ± 0.06	0.63 ± 0.13	0.84 ± 0.06
Chemical, protein target and cytotoxicity	0.82 ± 0.05	0.77 ± 0.10	0.86 ± 0.05

The vast majority of models passed the *y*-validation test (*i.e.* having a one-tailed *t*-test *p*-value <0.05), except for four out of the 100 models built using protein target descriptors only. These four models had CCR values <0.55, and as such were not significantly better than models built using scrambled data. The performance statistics of these models have not been removed from the final results to prevent unduly exaggerating the average performance of the protein target only models.

Considering models using only a single data domain, the mean CCR, sensitivity and selectivity values for the models built using chemical data alone were 0.78 (SD of 0.05), 0.72 (0.11) and 0.84 (0.06) respectively. The same metrics for the models built using solely protein target descriptors were 0.67 (0.06), 0.56 (0.12) and 0.78 (0.06); those for the models built using only cytotoxicity data were 0.67 (0.06), 0.40 (0.15) and 0.93 (0.03). For those predictive models built using dual data domains, the mean CCRs, sensitivities and selectivities respectively were: for the dual chemical and protein target model, 0.79 (0.05), 0.74 (0.12) and 0.86 (0.05); for the dual chemical and cytotoxicity model, 0.80 (0.06), 0.74 (0.12) and 0.86 (0.05); and for the dual protein target and cytotoxicity model, 0.73 (0.06), 0.63 (0.13) and 0.84 (0.06). The performance metrics of the models built from the complete tripartite dataset were a mean CCR of 0.82 (0.05), a mean sensitivity of 0.77 (0.10), and a mean selectivity of 0.86 (0.05).

The most accurate predictions on average are those made by the model built from all three descriptor sets, having a mean CCR of 0.82. In contrast, those models built solely from either protein target or cytotoxicity data domains have the poorest mean CCRs of 0.67. This is consistent with the study of Sedykh *et al.*, who “found qHTS *in vitro* data for cell viability alone to be insufficiently accurate classifiers of *in vivo* acute lethal toxicity”.^26^ However, the performance of the model which uses both protein target and cytotoxicity data has a more respectable mean CCR of 0.73. This supports the central hypothesis of our study, in that the combination of heterogeneous data is a useful technique for improving the performance of toxicity classification models. Although the increase in CCR on increased data inclusion is evident, the size of the standard deviations indicates that the performance of the models varies considerably depending on the training-test data split employed.

It was investigated whether the ability of the models trained on different descriptor sets would vary in their ability to classify the compounds of marginal toxicity, discarded earlier, into moderately toxic and moderately nontoxic classes. We found that no models were able to perform this task well, no matter which descriptors were used (with unsatisfactory CCRs ranging from 0.50 to 0.55).

**Fig. 4:**
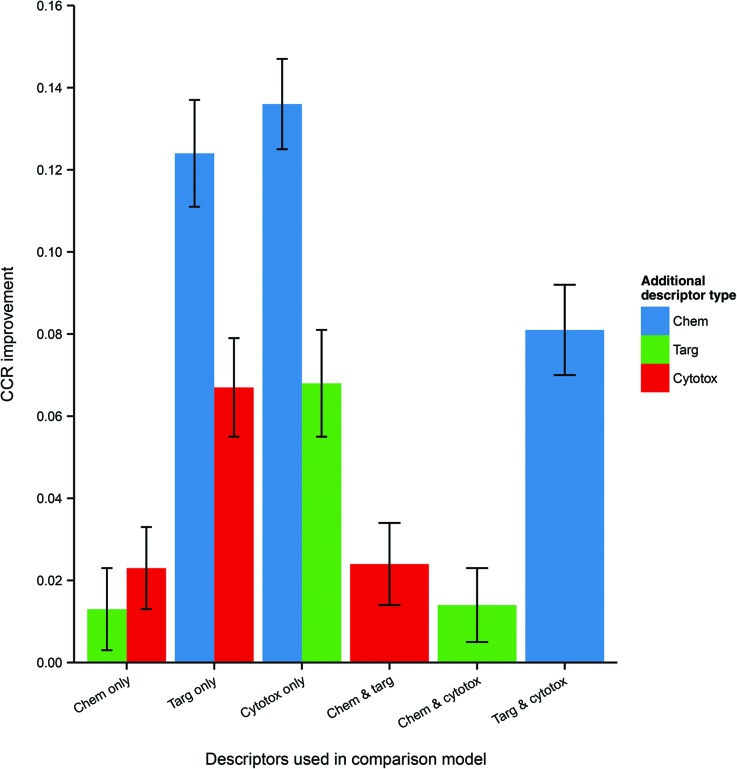
Mean CCR improvement on addition of further heterogeneous descriptors to models trained and tested on the same data. Here error bars represent the standard error in the value of the mean. Chemical data gives the biggest improvement (where originally absent), but it is clear that protein target and cytotoxicity data also improve performance when included. Abbreviations: as for Fig. 3.

Because the measured performance of a model is highly dependent on the split between training and testing data, in order to isolate and measure the effect of data integration it is necessary to remove the variability caused by different data splits. Therefore, the average differences in performance between models, trained and tested on the same data split, but built using different combinations of data domains were calculated and are given in Table 3 and visualized in Fig. 4.

**Table 3:** Differences in predictive performance on integrating further data domains. The CCR improvements refer to the increase in performance of models using the given descriptor set, compared with the models trained and tested on the same data but using the comparison descriptor set. The *p*-value is calculated using a two-tailed *t*-test with the null hypothesis that there is no difference in performance between models using the different descriptor sets

Descriptor set	Comparison descriptor set	CCR improvement (mean ± SE)	*p*-Value
Chemical and protein target	Chemical only	0.013 ± 0.005	8.1 × 10^–3^
Chemical and protein target	Protein target only	0.124 ± 0.005	2.5 × 10^–33^
Chemical and cytotoxicity	Chemical only	0.023 ± 0.005	3.5 × 10^–5^
Chemical and cytotoxicity	Cytotoxicity only	0.136 ± 0.007	8.1 × 10^–43^
Protein target and cytotoxicity	Protein target only	0.067 ± 0.006	5.2 × 10^–18^
Protein target and cytotoxicity	Cytotoxicity only	0.068 ± 0.006	4.9 × 10^–18^
Chemical, protein target and cytotoxicity	Chemical and protein target	0.024 ± 0.005	8.7 × 10^–6^
Chemical, protein target and cytotoxicity	Chemical and cytotoxicity	0.014 ± 0.005	2.3 × 10^–3^
Chemical, protein target and cytotoxicity	Protein target and cytotoxicity	0.081 ± 0.006	8.1 × 10^–26^

It is seen that, within a 95% confidence interval, inclusion of each of the three domains gives a performance improvement. The greatest improvement, of 13.6 CCR percentage points, is seen when including chemical data – which is to be expected, given the general utility of simple QSAR models and the strong performance of the chemistry-only model. However, there is also an evident improvement when including cytotoxicity and protein target information even as a third descriptor domain, with modest gains of 1.4 and 2.4 CCR points respectively in those cases. These results indicate that, for a given dataset, integration of heterogeneous data domains improves the performance of the model built using that dataset.

A further analysis of the effect of data domain on model performance – but using the area under the receiver operating characteristic (ROC) curve as the metric – is given in the ESI.†

### Effect of data domains on model coverage

**Fig. 5:**
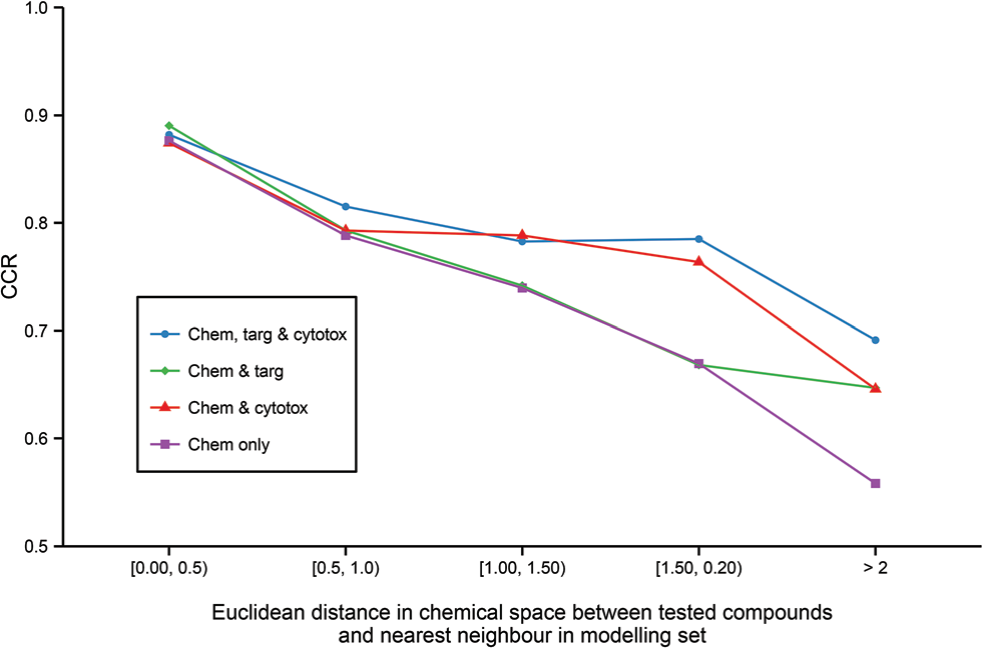
Applicability domains of each modelling technique. The figure illustrates the enhanced extrapolation into chemical space facilitated by the inclusion of heterogeneous descriptors. While all models give similar CCR performance of >0.85 when tested on the chemical compounds that are the most similar to the training data, the models built on chemical descriptors only give the worst performance on less similar chemicals with a CCR of <0.6 on the least similar chemicals. In contrast, the models built from all three data domains are the best performing on the molecules that are the most dissimilar to the training set. Abbreviations: as for Fig. 3.

Although predictive bioactivity models may be capable of formally classifying any compound, it is widely acknowledged that a defined applicability domain (AD) is mandatory in order to prevent such models groundlessly extrapolating into unexplored chemical space. To explore how the integration of heterogeneous descriptors into regular models may affect their ADs, for all models utilizing chemical descriptors, the accuracy of prediction against distance to the nearest neighbor in the modelling set in chemical space across all models utilizing chemical descriptors (Fig. 5) was compared.

It can be observed that all the models have similar strong predictive accuracy of >0.85 CCR on molecules very close to the modelling set. However, while all models become progressively less accurate the less similar the tested compound is to the training data, the models built with additional heterogeneous descriptors outperform the simple chemical model in their ability to accurately classify compounds further out into chemical space not covered in the training set. Indeed, Fig. 5 shows that the strong comparative performance of the tripartite model illustrated in Table 2 and Fig. 3 is largely due to the model's improved ability to successfully classify a broader range of molecules than the other models, rather than simply being a more accurate classifier with a similar applicability domain.

### Model interpretation

The use of cytotoxicity and especially protein target descriptors in predictive toxicity models provides the additional advantage of improving the interpretability of resultant models. Through an analysis of which descriptors are most important in successful classification, influences on the mode-of-action of the toxicity can be uncovered.

The random forest algorithm implemented in R^40^ allows for the extraction from a model of variable importance, range-scaled to between 0 and 100. These importance figures were extracted from the 100 tripartite models constructed in this study in order to investigate what additional information about toxicity mechanism might be gleaned (see ESI† for more). The top 10 most important descriptors from each domain, ordered by average importance over tripartite models, are given in Table 4. These features, being important to the classification of molecules, can be suspected of involvement in the processes leading to organism-level toxicity. In contrast to the esoteric meaning of the important chemical descriptors, the specified protein names are far more easily interpreted.

**Table 4:** Top 10 highest average importance descriptors from each data domain, as measured in the tripartite models. Overall, chemical descriptors are most important – although not all important descriptors are chemical. Protein names and cell lines are more interpretable than individual chemical descriptors

MOE chemical descriptors			Protein target descriptors		Cytotoxicity descriptors	
Ave. imp.	Code	Description	Ave. imp.	Protein name	Ave. imp.	Cell line and dose
90.6	a_ICM	Atom information content (mean)	35.5	Heat shock protein HSP 90-beta	33.5	Hek293, 92 μM
86.2	density	Molecular mass density	31.1	Endothelin B receptor	30.7	N2A, 92 μM
47.9	KierA3	A Kappa Shape index	29.3	KiSS-1 receptor	26.9	H4lle, 92 μM
47.0	SlogP_VSA9	A subdivided surface area descriptor	29.1	Carbonic anhydrase 14	25.9	jurkat, 46 μM
44.1	GCUT_PEOE_0	An adjacency and distance matrix descriptor	29.0	Tyrosine-protein phosphatase non-receptor type 7	21.3	jurkat, 92 μM
40.5	GCUT_PEOE_2	An adjacency and distance matrix descriptor	28.2	Plasminogen activator inhibitor 1	21.3	MRC5, 92 μM
39.2	PEOE_VSA-2	A partial charge descriptor	28.1	Somatostatin receptor type 4	21.2	SKNSH, 92 μM
38.1	BCUT_SLOGP_3	An adjacency and distance matrix descriptor	23.8	E3 ubiquitin-protein ligase Mdm2	16.6	N2A, 46 μM
34.0	GCUT_SMR_0	An adjacency and distance matrix descriptor	22.0	Multidrug resistance protein 1	15.5	SHSY, 46 μM
30.8	PEOE_RPC+	A partial charge descriptor	20.9	C5a anaphylatoxin chemotactic receptor 1	14.0	H4lle, 46 μM

For example, the two most-important targets are HSP 90-beta and the endothelin receptor type B. Heat shock proteins are essential for cellular homeostasis under stress conditions and can even interact with the programmed-cell death system.^41^ The endothelin receptor is suspected of contributing to the pathogenesis of myocardial infarction, bronchial asthma, renal failure amongst other diseases;^42^ endothelin itself is a potent vasoconstrictor and is implicated as an important factor in the development of cardiovascular disease,^43^ and has been shown to cause small intestinal mucosal damage in rats through significant hemorrhagic and necrotic lesions.^44^ A further enriched target involved in vascular system hemostasis is plasminogen activator inhibitor 1, elevated levels of which are associated with an increased risk of arterial thrombotic events, while deficiencies result in bleeding disorders.^45^ Patients with a plasminogen activator inhibitor-1 deficiency suffer from frequent bleeding episodes, while its increased expression has been shown to lead to numerous kidney diseases.^46^ Additionally, E3 ubiquitin-protein ligase is known to bind to a tumor suppressor, and abnormal regulation of E3 ligases has been shown to contribute to cancer development.^47^

Analysis of these protein target descriptors, several of which are implicated in a range of pathologies, affords much more scope for further mode-of-action investigation than their chemical counterparts. The presence of various different cell lines and doses in Table 4 indicate that these are each providing somewhat complementary information. As toxicity may arise for a range of reasons, beyond chemical reactivity or simple target binding, so we find that the most important cytotoxicity descriptors have comparable importance to the most important protein target descriptors. Further descriptor analysis is provided in the ESI,† including the example of *N*,*N*′-di-*sec*-butyl-*p*-phenylenediamine, which is only correctly identified as toxic when cytoxocitity descriptors are included. Such examples are to be expected, given the heterogeneous mechanisms by which toxicity can occur.

To further illustrate the potential benefits of descriptor analysis afforded through using heterogeneous descriptors, a subset of the dataset was extracted (defined as all molecules that were classified correctly every time they were used to test a tripartite model). Of these molecules, phenacyl chloride (the active ingredient in the original formulation of Mace spray) was successfully predicted to be toxic although it possesses five non-toxic neighbors in chemical space within a Euclidean distance of 0.82 and hence represents a molecule that is not expected to be easily classified correctly in this space.

Phenacyl chloride is chemically similar to these five other compounds, yet is consistently able to be distinguished and correctly classified as toxic by the tripartite model. In contrast, no simple chemistry-only models correctly classified phenacyl chloride as toxic. We can therefore infer that the combination of the protein target and cytotoxicity descriptors enabled the tripartite model to detect phenacyl chloride's toxicity. For each of these non-toxic neighbors, the five protein target descriptors which display the largest numerical increase going from the compound in question to phenacyl chloride – and which are therefore implicated as being more likely to interact with the toxic compound than the non-toxic – are given in Table 5. Amongst the most frequently found protein targets are the histamine H_2_ receptor, antagonists of which have been shown to induce neurotoxic convulsions in mice,^48^ and macrophage metalloelastase which is associated with inflammatory diseases such as aneurysms, cancers and chronic pulmonary inflammatory diseases.^49^ Given the frequency of their appearance, and their known involvement in toxic outcomes, the assumption that these targets may be involved in the mode of action of phenacyl chloride is a good starting point for further investigation. Such interpretations, linking descriptor analysis to the experimental results of exposure, are made possible through the integration of target information into the predictive model.

**Table 5:** Comparison of a toxic compound that was classified with 100% accuracy by the tripartite models with its 5 nearest non-toxic neighbours in chemical space that were similarly perfectly predicted. These compounds give an example of a toxicity which is best predicted using non-chemical descriptors. The implicated protein targets are those whose predicted interaction likelihood exhibit the largest numerical increase in value going from the nontoxic to the toxic compound. This indicates an increased likelihood of interaction with the toxic compound in comparison to the nontoxic, giving clues towards the toxic mode of action. Protein targets which appear three or more times are italicised

	Structure and name	Implicated protein targets
Toxic compound	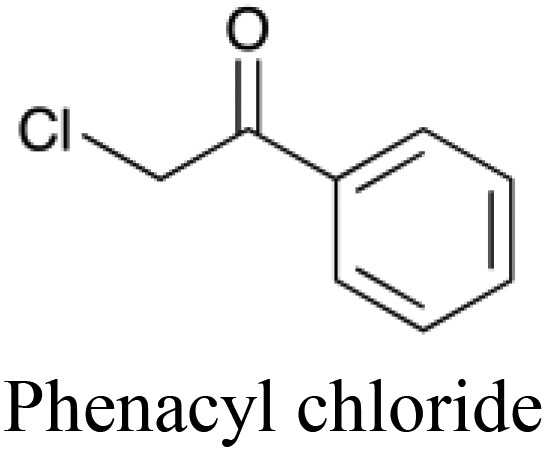	n/a
Non-toxic neighbors	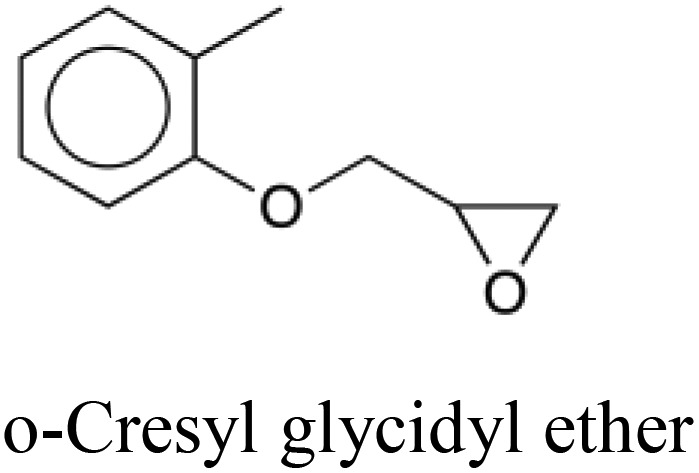	1. Protein kinase C zeta type
		2. *Vesicular acetylcholine transporter*
		3. *Macrophage metalloelastase*
		4. Bombesin receptor subtype 3
		5. Heat shock protein HSP 90 beta
	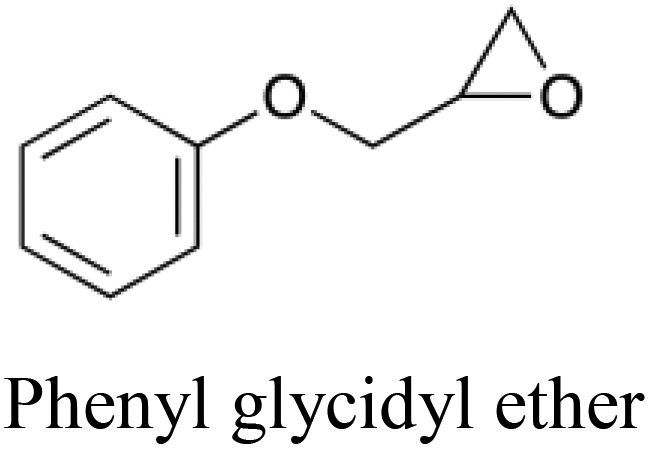	1. *Vesicular acetylcholine transporter*
		2. Somatostatin receptor type 4
		3. Protein kinase C zeta type
		4. Chymase
		5. *Macrophage metalloelastase*
	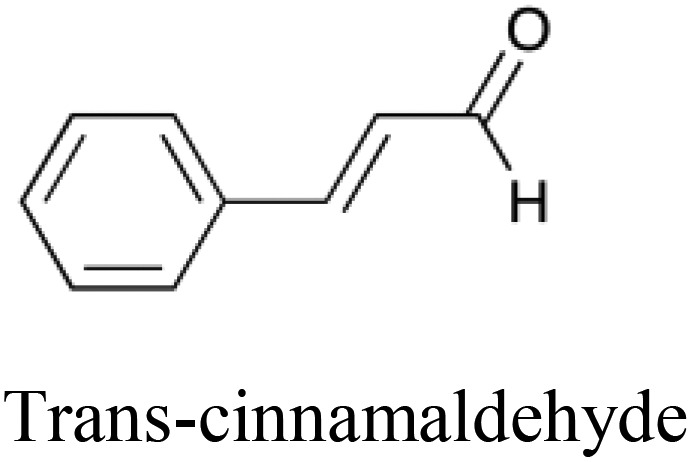	1. *Vesicular acetylcholine transporter*
		2. *Histamine H*_*2*_*receptor*
		3. Multidrug resistance protein 1
		4. *Macrophage metalloelastase*
		5. Sodium and chloride dependent glycine transporter 1
	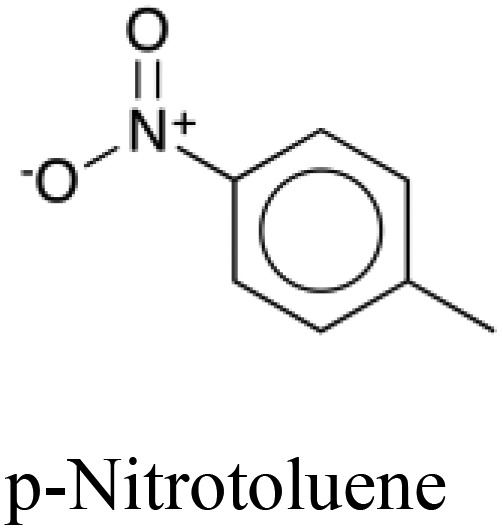	1. *Histamine H*_*2*_*receptor*
		2. Bombesin receptor subtype 3
		3. Heat shock protein HSP 90 beta
		4. Caspase 8
		5. KiSS 1 receptor
	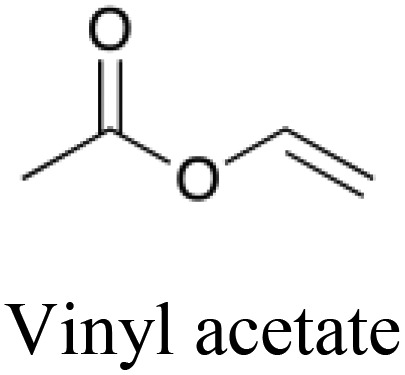	1. *Histamine H*_*2*_*receptor*
		2. *Vesicular acetylcholine transporter*
		3. *Macrophage metalloelastase*
		4. Sodium and chloride dependent glycine transporter 1
		5. Chymase

## Conclusions

We have investigated the change of average external predictive power for toxicity classification models using various combinations of (i) chemical descriptors (derived from compounds’ structures and computed physiochemical properties), (ii) protein target descriptors (derived from a validated *in silico* predictive model trained on the wealth of experimental bioactivity available in ChEMBL), and (iii) cytotoxicity descriptors (derived from the National Toxicology Program's qHTS studies on 13 cell lines). This study suggests that, although non-traditional descriptor domains have limited utility in the building of predictive models by themselves, the accuracy of external prediction is enhanced through the use of multiple heterogeneous descriptor types along with the chemical descriptors used in conventional QSAR approaches.

The notion that more, varied bioactivity information should furnish more accurate toxicity predictions is an intuitive one. However, the evidence we have provided for the increased predictive accuracy of models built on a tripartite descriptor set, along with the utilization of an *in silico* approach to the provision of target-affinity information, should encourage the use of this integrative technique in future predictive toxicological studies both for its predictive power and demonstrated practicality. We have further illustrated that a heterogeneous model has a broader applicability domain, being able to correctly classify chemically dissimilar compounds that a solely chemistry-based model cannot, and provides more interpretable results than comparable homogenous models. Finally, we have found that including different data domains affords models which display differing degrees of sensitivity and selectivity imbalance (for example, the models built using only cytotoxicity descriptors have very strong selectivity yet poor sensitivity; models built using chemical descriptors have more balanced sensitivity and selectivity). This is of particular relevance to practical applications of this methodology: depending on whether efficacy or safety is the first priority, certain descriptor domains may be more useful than others.

The greatest limitation of this study is that only 367 structures were utilised in the modelling and testing runs; future work will require a larger dataset, which will amongst other benefits facilitate a more precise measurement of the models’ applicability domains. Initial explorative attempts to classify the large number of compounds of marginal toxicity, currently not utilised, were not successful.

In future work, an exploration of how dimensionality reduction might best be applied to a heterogeneous dataset would be valuable. Simple co-correlation analysis, while computationally facile, is unlikely to produce an optimum subset of descriptors from each domain. In addition, the methodology behind the selection of qHTS cell lines has not been considered in this study. An optimally-selected qHTS dataset, comprising assays identified as highly predictive of *in vivo* toxicity, would afford more predictive cytotoxicity descriptors, both in isolation and in combination with other descriptor sets.

Nonetheless, as has been demonstrated in the present study, with the advent of predictive, validated *in silico* methods for predicting protein–ligand affinities and phenotypic outcomes, the feasibility and utility of constructing toxicological prediction models based on heterogeneous bioactivity data is increasing. The inclusion of cytotoxicity data has been shown to provide sufficient increases in accuracy and interpretability to justify the added complexity of acquiring the experimental data. The inclusion of protein target data, generated *in silico* affords predictive models with information relevant to the complex metabolic and signalling pathways with which the compounds interact *in vivo*, subsequent to initial exposure. Unless metabolism can be reliably accounted for within computational toxicology, *in vivo* testing will still be necessary to identify metabolites for future profiling.^3^

It is therefore hoped that the methodology of creating integrative models such as those investigated in this study will be further explored and improved, including investigating the use of further data domains such as pathways and metabolites. Such modelling approaches may eventually develop to become a powerful tool in the drug discovery and toxicity screening pipelines.

## Supplementary Material

Supplementary information
