# Supplementary material for: Improving the prediction of organism-level toxicity through integration of chemical, protein target and cytotoxicity qHTS data
Source: Toxicol Res (Camb). 2016 Mar 3;5(3):883–94. doi: 10.1039/c5tx00406c (PMC6062365; doi:10.1039/c5tx00406c)
Supplement: Supplementary file 1 [file TX-005-C5TX00406C-s001.pdf]

Electronic Supplementary Material (ESI) for Toxicology Research.
This journal is © The Royal Society of Chemistry 2016

Improving the Prediction of Organism-level Toxicity through Integration of Chemical, Protein Target and Cytotoxicity qHTS Data

Electronic Supplementary Information

Chad H. G. Allen [1], Alexios Koutsoukas [1], Isidro Cortes-Ciriano [2], Daniel S. Murrell [1], Thérèse E. Malliavin [2], Robert C. Glen [1] and Andreas Bender [1]*

1. Centre for Molecular Informatics, Department of Chemistry, Lensfield Road, Cambridge CB2 1EW, UK
2. Unite de Bioinformatique Structurale, Institut Pasteur and CNRS URA 2185, Structural Biology and Chemistry Department, Paris, France

***Corresponding author:**

Centre for Molecular Informatics

Department of Chemistry

Lensfield Road

Cambridge CB2 1EW

UK

Telephone: 01223 762983

E-mail: ab454@cam.ac.uk

Table of Contents

Supplementary Methods **3**

Standardization *3*

Descriptor selection *3*

Supplementary Analysis **5**

Dataset chemistry *5*

Model performance between data domains *5*

Descriptor importance *5*

Receiver operating characteristic (ROC) curves *6*

Supplementary Tables **9**

Table S1 *9*

Table S2 *10*

Table S3 *11*

Supplementary Figures **12**

Figure S1 *12*

Figure S2 *13*

Figure S3 *14*

Figure S4 *15*

Figure S5 *16*

Supplementary Methods

Standardization

Before generation of chemical and protein target descriptors, all structures were standardized using ChemAxon's Standardizer (version 5.11.5, 2013, www.chemaxon.com). The optional steps selected were retention of only the largest fragment of fragmented compounds, neutralizing species, and canonicalizing tautomers.

Descriptor selection

In order to reduce the total number of descriptors, and to ensure similar numbers of descriptors from each data type, individual maximum correlation cutoffs were derived for each data domain.

The cytotoxicity descriptors, being the fewest, were allocated a maximum correlation cutoff of 0.90, *i.e.* for every pair of descriptors with a pairwise correlation of over 0.90, the descriptor with the highest average correlation to the rest of the descriptors is removed. This routine applied to the whole descriptor set was found to leave 55 cytotoxicity descriptors.

For the chemical and protein target descriptor sets, starting with 0.90, a cutoff was applied to the descriptors and the number of remaining descriptors was counted. If the number was less than 66, which is within 20% of the number of cytotoxicity descriptors retained with a cutoff of 0.90, this cutoff was chosen. Otherwise, the cutoff was lowered by 0.05 and the number of resulting descriptors counted again. In this way, correlation cutoffs of 0.75 for chemical and 0.60 for protein target descriptors were chosen. The relative sizes of these cutoffs reflect both the original numbers of descriptors present from each domain (*i.e.* 182 cytotoxicity

descriptors, 192 chemical descriptors, and 477 protein target descriptors) as well as the degree of correlation within a data type.

These optimum cutoffs were derived in advance from the entire dataset. This avoided the computational burden of deriving individual correlation cutoffs for each of the 100 training sets employed, as only approximately equal numbers of descriptors are required. However, the cutoffs were only applied once the validation set had been set aside, and so the selection of which descriptors to discard was determined by the correlations between the descriptors in the modelling set.

Downsampling

Because of the imbalance in class sizes (275 nontoxic compounds, in comparison with 92 toxic) among the dataset employed, it was necessary to use a downsampling procedure to better balance the classes within each modelling set.

In the descriptor space defined by the chemical descriptors, the Euclidean distances between each pair of toxic and nontoxic chemicals was calculated. Only those nontoxic molecules closer than $d - 0.5\ \sigma$ to a toxic compound in chemical descriptor space (where d is the average and σ the standard deviation of interclass distances) were retained to train the model.

In this way, those nontoxic molecules easily identifiable through a naïve chemical similarity screen were excluded from model building.

Supplementary Analysis

Dataset chemistry

The dataset was analysed for common Marcko frameworks. These are given in Table S1.

It can be seen that the most common framework by far represents simple aromatic molecules, accounting for over 100 structures. Only eight other frameworks were seen more than once, of which all but two were also aromatic.

Model performance between data domains

Figure S1 illustrates the varying performances of differently constructed models, projected onto the 2D space described by the first two principal components in tripartite space for ease of comparison, and how they compare to the performance of the superior tripartite model.

A number of molecules are poorly predicted by two of the single domain models but well predicted by the tripartite model. In 4, the toxic chemical clearly distinguishable at the top right corner of the plots, it is only correctly identified as toxic when cytotoxicity descriptors are included. We may therefore conclude that this compound's toxicity is encoded only in these descriptors.

It is therefore observed that the tripartite model is not simply an averaging of the three single-data models, but rather an integration and improvement – taking into account the most relevant information from each domain..

Descriptor importance

It can be seen in Figure S2 that the distribution of protein targets is less skewed than the other two domains. This implies that many different proteins may be involved in toxicity

pathways, and that therefore a large panel of protein targets may be required for best results (though it may also be indicative of mutual correlations in this data domain).

Receiver operating characteristic (ROC) curves

ROC curves are a means of visualising the trade-offs inherent in the choice of cutoff used to transform the real-valued output of a scoring classifier into a binary class prediction. A ROC curve plots a cutoff-parameterised curve in the plane described by the true positive rate (*i.e.* the sensitivity) and the false positive rate. In this way, the predictive performance of a classifier may be visualised independent of the cutoff chosen to be used. [1] The area underneath the ROC curve (AUC) is therefore a general measurement of predictive power, complementary to CCR, sensitivity and selectivity.

The modelling procedure outlined in the main paper was repeated in full, and the AUC was this time measured as the main performance metric. The results are given in Tables S2 and S3, and illustrated by Figures S3 and S4.

The pattern of improving performance with further integration of descriptor domains is again evident using this metric (*e.g.* an average increase in AUC between models trained and tested on the same compounds of 0.053 ± 0.005 on adding cytotoxicity descriptors to chemical and protein target descriptors, and an average increase of 0.125 ± 0.007 on adding protein target descriptors to cytotoxicity descriptors), with the exception that using this metric no statistically meaningful change is observed when adding protein target descriptors to models which already use chemical descriptors.

Figure S5 displays ROC curves for a selection of models, generated in the ROCR package for R. It can be seen that the models built using protein target descriptors alone show more variation in performance when compared to the other ROC curves shown. The most

consistent performances are seen from the model using integrated descriptor domains. Another striking feature is the skew towards the left-hand axis of the models build using cytotoxicity alone: this represents conservative predictive behaviour, making few (mainly accurate) positive predictions. This dovetails with the high-selectivity, low-sensitivity behaviour exhibited by such models in the main paper, and suggests that cytotoxicity descriptors may possibly only be identifying certain toxicities rather than having a broad applicability.

Supplementary Reference

1. T. Sing, O. Sander, N. Beerenwinkel and T. Lengauer, *Bioinformatics*, **21**, 3940-3941.

Supplementary Tables

Table S1

Rank	Occurrences	Framework SMILES	Framework structure
**1**	101	c1ccccc1	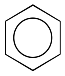
**2**	6	c1ccc(Cc2ccccc2)cc1	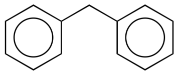
**= 3**	5	c1ccc2c(c1)OCO2	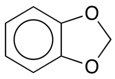
**= 3**	5	c1ccncc1	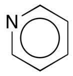
**= 3**	5	C1CO1	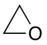
**= 6**	3	c1ccc(-c2ccccc2)cc1	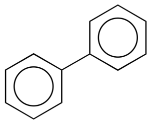
**= 6**	3	C1CCCCC1	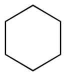
**= 6**	3	c1ccc2ccccc2c1	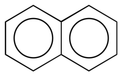
**= 6**	3	c1ccc(C2CO2)cc1	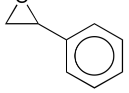

The most common Marcko frameworks in the dataset. Of the 367 structures, 232 could be reduced to Marcko frameworks. Any frameworks occurring more than twice in the dataset are displayed; these frameworks correspond to 134 structures (58% of the frameworks, or 37% of the structures).

Table S2

Descriptor domains	AUC (mean ± SD)
Chemical only	0.84 ± 0.05
Protein target only	0.73 ± 0.07
Cytotoxicity only	0.67 ± 0.07
Chemical and protein target	0.83 ± 0.05
Chemical and cytotoxicity	0.85 ± 0.05
Protein target and cytotoxicity	0.83 ± 0.05
Chemical, protein target and cytotoxicity	0.85 ± 0.05

Means and standard deviations of the area under the ROC curve for models built using each combination of data. With this metrics, the improvement on integration of further data domains is less pronounced – especially when compared to the performance of the models built using chemical descriptors alone. Once again the standard deviations indicate strong variability of performance across different selections of training and test data.

Abbreviations: AUC, area under the ROC curve.

Table S3

**Descriptor set**	**Comparison descriptor set**	**AUC change (mean ± SE)**	***p*-value**
Chemical and protein target	Chemical only	−0.004 ± 0.002	7.3×10^{-3}
Chemical and protein target	Protein target only	0.104 ± 0.007	$< 2.2 \times 10^{-16}$
Chemical and cytotoxicity	Chemical only	0.010 ± 0.002	4.7×10^{-6}
Chemical and cytotoxicity	Cytotoxicity only	0.177 ± 0.007	$< 2.2 \times 10^{-16}$
Protein target and cytotoxicity	Protein target only	0.067 ± 0.005	$< 2.2 \times 10^{-16}$
Protein target and cytotoxicity	Cytotoxicity only	0.125 ± 0.007	$< 2.2 \times 10^{-16}$
Chemical, protein target and cytotoxicity	Chemical and protein target	0.015 ± 0.002	1.7×10^{-10}
Chemical, protein target and cytotoxicity	Chemical and cytotoxicity	0.0006 ± 0.002	0.76
Chemical, protein target and cytotoxicity	Protein target and cytotoxicity	0.053 ± 0.005	4.8×10^{-16}

Differences in predictive performance on integrating further data domains. As in Table 3 of the main paper, here area under the ROC curve improvements refer to the increase in performance of models using the given descriptor set, compared with the models trained and tested on the same data but using the comparison descriptor set. The *p*-value is calculated using a two-tailed *t*-test with the null hypothesis that there is no difference in performance between models using the different descriptor sets. Using this metric, there is no significant difference made to performance on including protein target descriptors compared to using chemical descriptors only, or using chemical and cytotoxicity descriptors; however, integrating protein target descriptors with cytotoxicity descriptors yields an improvement of 0.125 ± 0.007 on cytotoxicity descriptors alone.

Abbreviations: AUC, area under the ROC curve.

Supplementary Figures

Figure S1

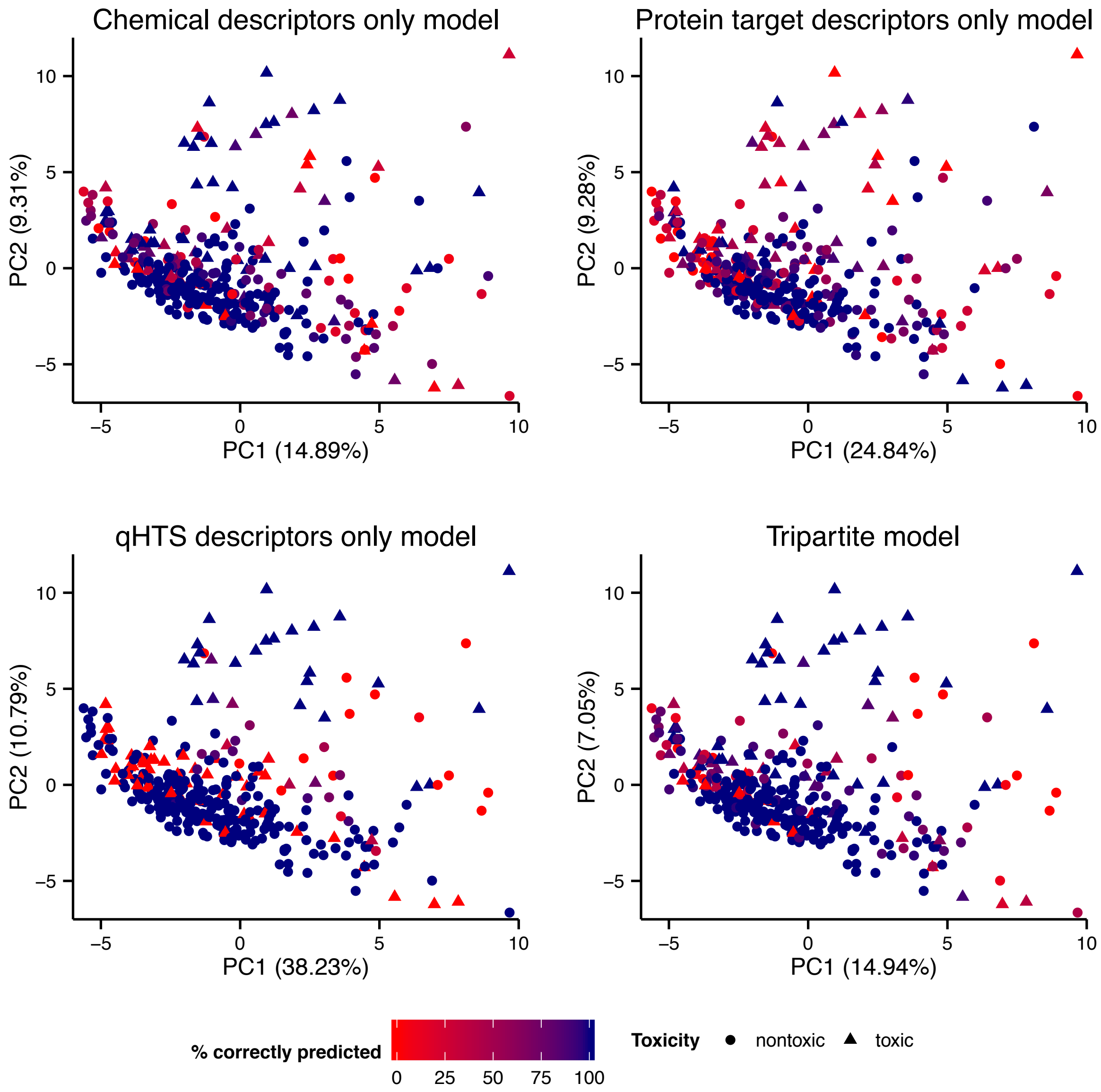

Performances of single domain and tripartite models mapped onto a section of the principal components analysis plot of the tripartite descriptor space. The two principal components plotted represent 22% of the total variance in tripartite space, and 16 outlier compounds are not visible within these axis limits.

Figure S2

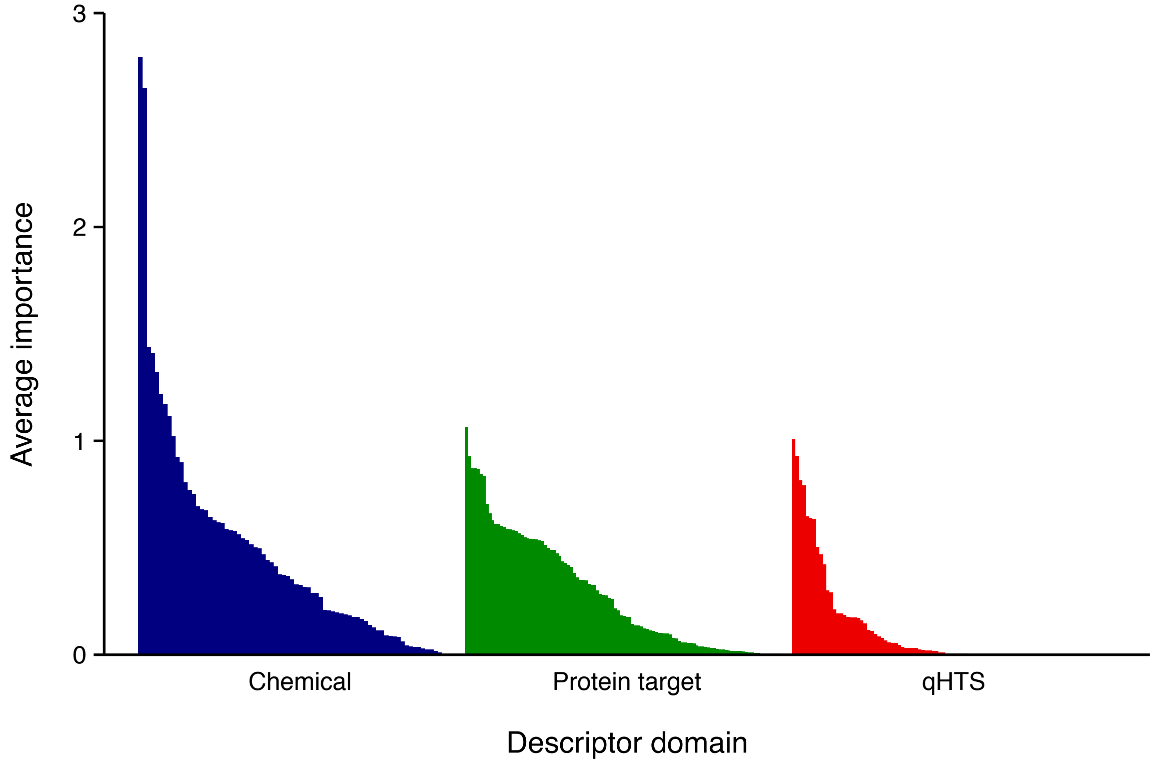

Plots of descriptor importance distributions for the three data domains.

Figure S3

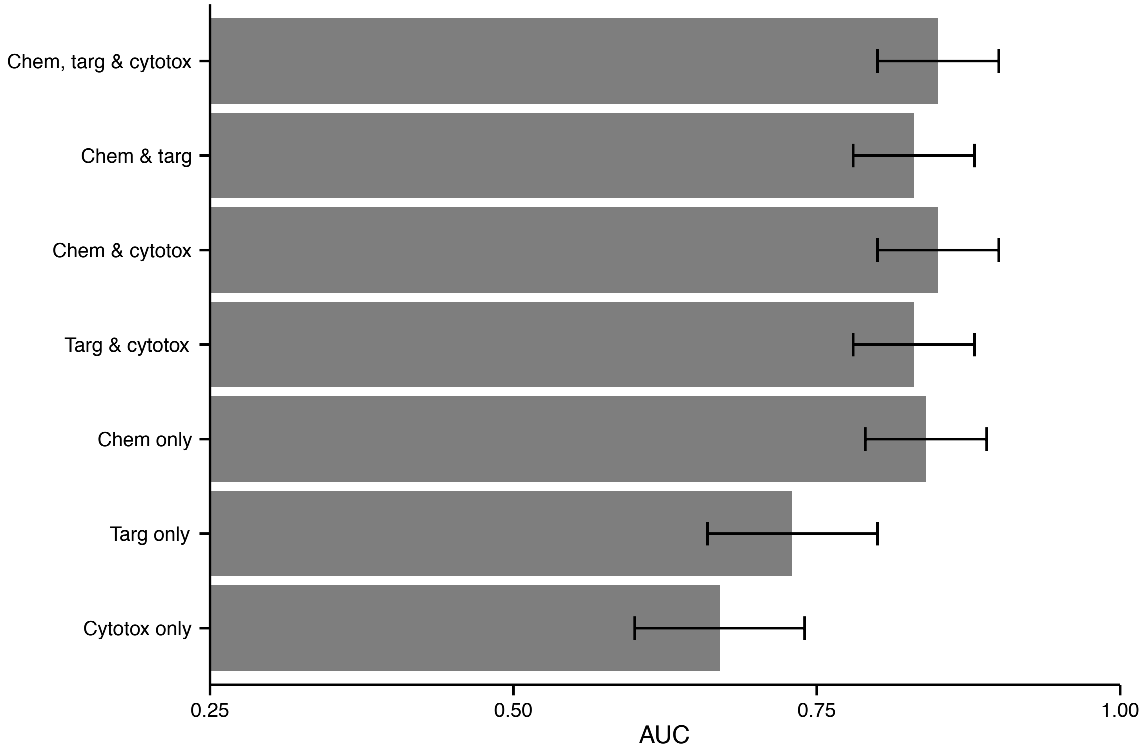

Performance distributions of predictive model build using each combination of descriptor domains, measured by area under the ROC curve. Here error bars are used to display the standard deviations in the performance distributions, illustrating the marked dispersal from the mean. Models including chemical descriptors tend to perform most strongly, and there is a pattern of increased performance on addition of chemical or cytotoxicity descriptors.

Abbreviations: AUC, area under the ROC curve; Chem, chemical descriptors; Targ, protein target descriptors; Cytotox, cytotoxicity descriptors.

Figure S4

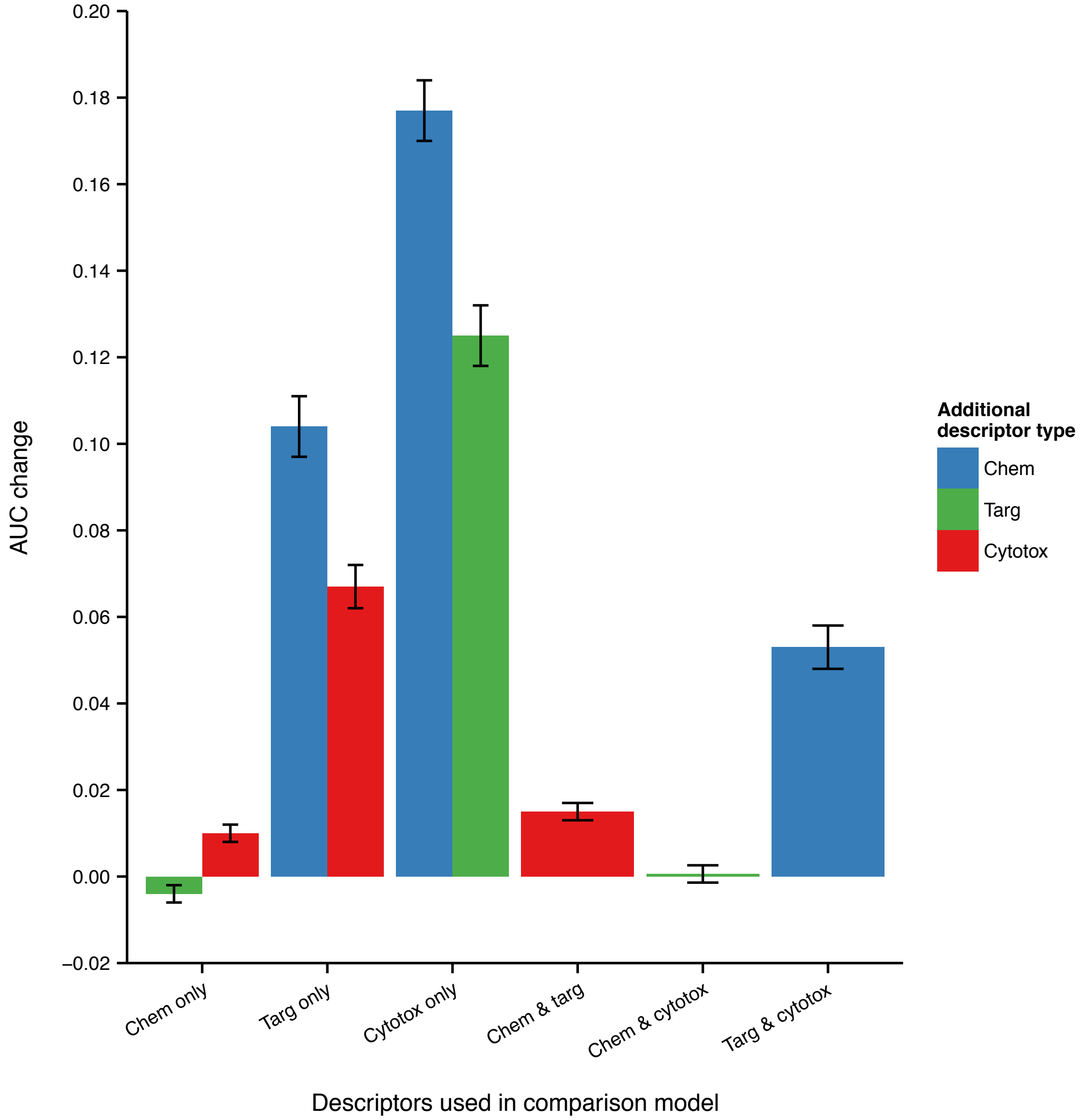

Mean change in the area under the ROC curve on addition of further heterogeneous descriptors to models trained and tested on the same data. Here error bars represent the standard error in the value of the mean. Chemical data still give the biggest improvement (where originally absent), but it is evident that protein target descriptors do not improve the ROC statistic when added to descriptor sets already containing chemical descriptors.

Abbreviations: as for Figure S3.

Figure S5

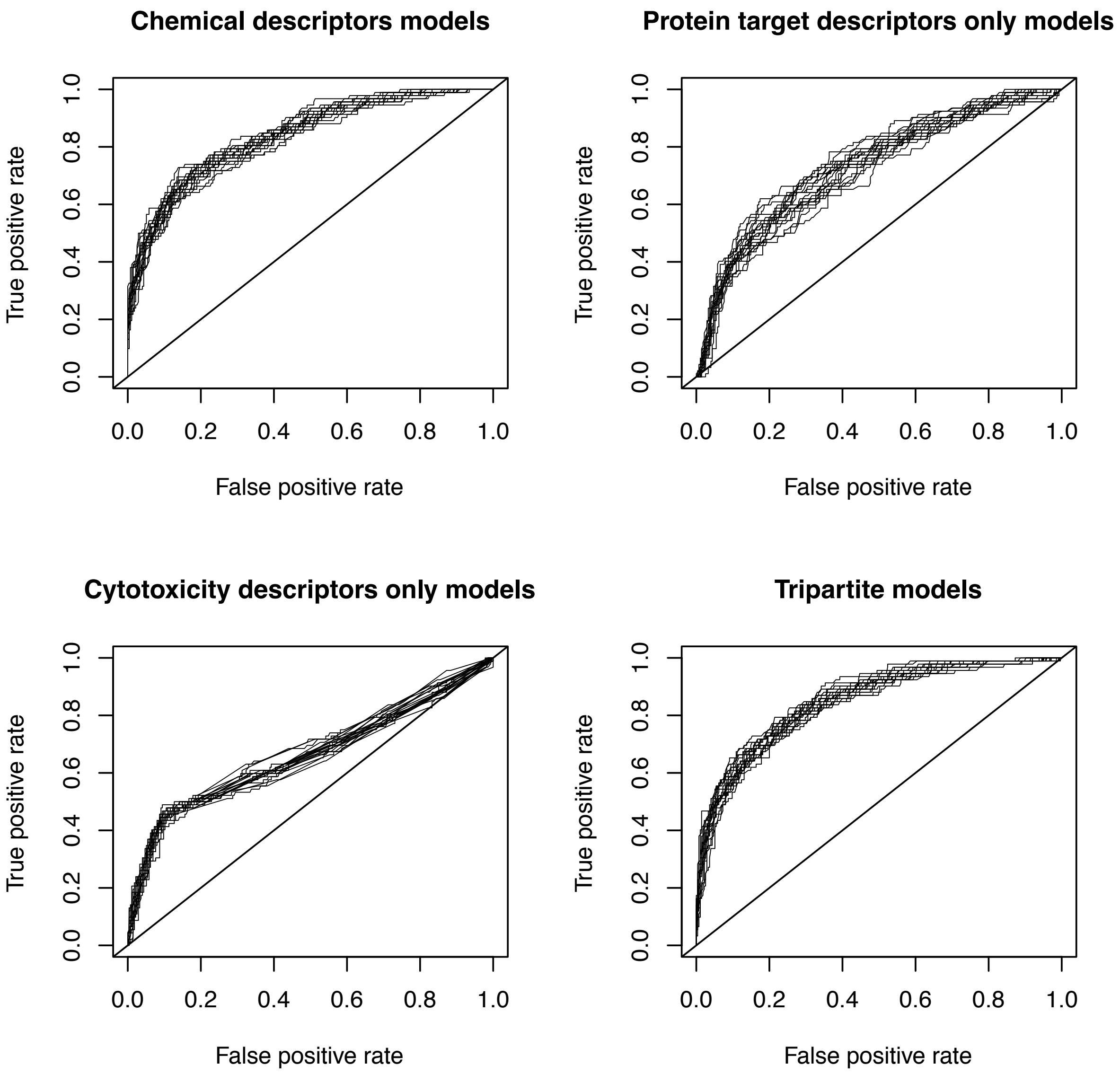

ROC curves for a selection of models. Each individual line on a plot corresponds to one of the 20 full cross-validation repeats, in which each each compound is predicted exactly once. All curves are roughly symmetrical, except for those representing models built using cytotoxicity descriptors only, which skews towards the left-hand axis. The curves representing models built using protein target descriptors only show the most variation in performance, while the curves representing models built using the full tripartite descriptor set exhibit least variation.
